# Intratumor Heterogeneity Correlates With Reduced Immune Activity and Worse Survival in Melanoma Patients

**DOI:** 10.3389/fonc.2020.596493

**Published:** 2020-12-04

**Authors:** Zhen Lin, Xianyi Meng, Jinming Wen, José María Corral, Darja Andreev, Katerina Kachler, Georg Schett, Xiaoxiang Chen, Aline Bozec

**Affiliations:** ^1^ Department of Internal Medicine 3—Rheumatology and Immunology, Friedrich-Alexander-University Erlangen-Nürnberg (FAU) and Universitätsklinikum Erlangen, Erlangen, Germany; ^2^ Deutsches Zentrum für Immuntherapie (DZI), Friedrich-Alexander-University Erlangen-Nürnberg (FAU) and Universitätsklinikum Erlangen, Erlangen, Germany; ^3^ Division of Biochemistry, Friedrich-Alexander-Universität Erlangen-Nürnberg, Erlangen, Germany; ^4^ Department of Rheumatology, Renji Hospital Affiliated to Shanghai Jiao Tong University School of Medicine, Shanghai, China

**Keywords:** immunomodulator, intratumor heterogeneity, The Cancer Genome Atlas, tumor infiltrating lymphocytes, melanoma

## Abstract

**Background:**

Human malignant melanoma is a highly aggressive, heterogeneous and drug-resistant cancer. Due to a high number of clones, harboring various mutations that affect key pathways, there is an exceptional level of phenotypic variation and intratumor heterogeneity (ITH) in melanoma. This poses a significant challenge to personalized cancer medicine. Hitherto, it remains unclear to what extent the heterogeneity of melanoma affects the immune microenvironment. Herein, we explore the interaction between the tumor heterogeneity and the host immune response in a melanoma cohort utilizing The Cancer Genome Atlas (TCGA).

**Methods:**

Clonal Heterogeneity Analysis Tool (CHAT) was used to estimate intratumor heterogeneity, and immune cell composition was estimated using CIBERSORT. The Overall Survival (OS) among groups was analyzed using Kaplan–Meier curves with the log-rank test and multivariate cox regression. RNA-seq data were evaluated to identify differentially expressed immunomodulatory genes. The reverse phase protein array (RPPA) data platform was used to validate immune responses at protein level.

**Results:**

Tumors with high heterogeneity were associated with decreased overall survival (p = 0.027). High CHAT tumors were correlated with less infiltration by anti-tumor CD8 T cells (p = 0.0049), T follicular cells (p = 0.00091), and M1 macrophages (p = 0.0028), whereas tumor-promoting M2 macrophages were increased (p = 0.02). High CHAT tumors correlated with a reduced expression of immunomodulatory genes, particularly Programmed Cell Death 1 (PD1) and its ligand PD-L1. In addition, high CHAT tumors exhibited lower immune Cytotoxic T lymphocytes (CTLs)-mediated toxicity pathway score (p = 2.9E−07) and cytotoxic pathway score (p = 2.9E−08). High CHAT tumors were also associated with a lower protein level of immune-regulatory kinases, such as lymphocyte-specific protein tyrosine kinase (LCK) (p = 3.4e−5) and spleen tyrosine kinase (SYK) (p = 0.0011).

**Conclusions:**

Highly heterogeneous melanoma tumors are associated with reduced immune cell infiltration and immune response activation as well as decreased survival. Our results reveal that intratumor heterogeneity is an indicative factor for patient survival due to its impact on anti-tumor immune response.

## Introduction

Melanoma is a highly heterogeneous disease with genetic and phenotypic diversity (1). The coexistence of cells with different phenotypic and molecular features within one tumor is named intratumor heterogeneity (ITH) (2). It is well known that a high number of clones, each harboring a set of various mutations, results in an exceptional level of intratumor heterogeneity in melanoma (2). Pro-tumorigenic properties, such as a tumor-promoting inflammatory milieu, and the resistance to immune destruction, at least in part, may be linked to intratumor heterogeneity (3). In melanoma, heterogeneity at the genomic and immunological level influences cancer progression and results in differential responses to therapy (4). The evaluation of intratumor heterogeneity of individual tumors as well as its impact on disease progression and therapeutic efficacy is therefore essential to overcome treatment challenges in melanoma.

It is believed that tumor initiation and progression result from dominant mutations within the original healthy cells, followed with a selection of malignant subclones (3). The Clonal Heterogeneity Analysis Tool (CHAT) is a collection of tools developed for tumor clonality analysis using high density DNA SNP array data and sequencing data (5). Using CHAT, it is possible to estimate cellular fractions for both somatic copy number alterations (sCNAs) and mutations, and to use their distributions to define the underlying clonal structure.

Tumor immune surveillance is crucial to inhibit carcinogenesis, tumor progression, and to maintain cellular homeostasis. A failure of this surveillance system is associated with poor survival (6). Nowadays, classical cancer therapies, such as chemotherapy, surgery or radiation are combined with the administration of immunomodulators, functional molecules that amplify the patient’s immune response to cancer (7). Notably, tumor infiltrating lymphocytes (TILs) are an important characteristic to define the therapeutic action and could be used to estimate the success rate of novel melanoma immunotherapeutic protocols (8). Moreover, molecular and genetic signatures of the immune cytolytic activity are related to immune-mediated cancer elimination, which critically impacts the survival of cancer patients (9). To pave the way for personalized cancer therapy, immunogenomic analysis of tumors can be used to predict the tumor immune microenvironment, clinical associations, as well as prognostic biomarkers, indicating the efficacy of cancer immunotherapy (10, 11).

Up to now, the relationship between intratumor heterogeneity and the immune microenvironment across a large cohort of melanoma patients has not been explored in depth. Thus, in the present study we aim to investigate the association of tumor heterogeneity with the composition of tumor-infiltrating immune cells, as well as immunomodulators, anti-tumor immune activity, and survival in melanoma.

## Materials and Methods

### Patient Cohort

By virtue of genomic data commons data portal provided officially by the Cancer Genome Atlas (TCGA) database (12) (https://cancergenome.nih.gov/), a total of 402 untreated patients with DNA single nucleotide variants (SNVs) and Copy number variation (CNV) data were included in the skin cutaneous melanoma cohort (SKCM) of TCGA Provisional cohort, of whom clinical information were available. The gene expression quantification data for TCGA cohort was downloaded through UCSC xena (13). The reverse phase protein array (RPPA) data was downloaded from The Cancer Proteome Atlas (TCPA), a user-friendly data portal developed to facilitate the access of cancer proteomics datasets to the broader research community (14).

### Clonal Heterogeneity Analysis Tool (CHAT)

Tumors arise from a single mutated cell that accumulates additional mutations as it progresses. These changes give rise to tumor subpopulations, which have the ability to divide and mutate further (15). This heterogeneity may give rise to clones that have an evolutionary advantage over the others within the tumor environment, and these clones may become dominant in the tumor over time (15). CHAT estimates cellular fractions for both sCNAs and mutations, and uses their distributions to define the macroscopic, clonal architecture of the tumor and the overall intra-tumor heterogeneity, based on the estimated number of clones (5, 16). To study the correlation between heterogeneity and overall patient survival, we divided the TCGA patients into the following groups: Low ITH (clones 1, 2 and 3), High ITH (clones 4, 5 and 6).

### Survival Analysis

A cohort of TCGA melanoma patients with available clinical data was subdivided based on the number of clones as calculated by the CHAT algorithm (1). Next, we calculated Kaplan–Meier survival curves for each group and tested the statistical relevance of the obtained differences between the groups by a Log-rank test. The overall survival (OS) analysis was performed by using the survival package available for R. Due to the fact that other clinical factors, such as gender and age can influence the survival outcome, it was necessary to verify, whether the differences identified in this study occur independently of these parameters. Therefore, a multivariate cox regression analysis was performed in which gender, patient age (at the time of diagnosis), and clinical stage were incorporated as additional factors.

### CIBERSORT Estimation

CIBERSORT, a bioinformatics algorithm to calculate immune cell compositions based on gene expression profiles, was used to estimate the type of tumor-infiltrating immune cells (17). Immune cell fraction data were downloaded from TCIA database (https://tcia.at/home) (18). Proportions of 22 immune cells were compared between CHAT high and low tumors using the same cut-off for survival analysis in the TCGA cohort.

### Single-Sample Gene Set Enrichment Analysis (ssGSEA)

Single sample GSEA (ssGSEA) calculates a gene set enrichment score for each sample as the normalized difference in empirical cumulative distribution functions of gene expression ranks inside and outside the gene set (19). The Molecular Signatures Database (MSigDB) BIOCARTA collection was scored using ssGSEA as implemented in the GSVA R package (20).

### Statistical Analysis

Log-rank test and Cox proportional hazard analysis were used to compare the survival distribution between low and high groups calculated according to CHAT. Moreover, receiver operating characteristic (ROC) analysis was performed to evaluate predictive prognostic effect. Pearson correlations were calculated based on expression levels of immune cell fraction, and was plotted afterwards. Immune cell fraction comparison was analyzed using the Wilcoxon signed-rank test and gene expression comparison was analyzed using Student’s t test. In all analyses, A probability value of p <0.05 was considered statistically significant (*p <0.05; **p <0.01; ***p <0.001; ****p <0.0001). All statistical analyses and plots were generated using R version 3.6.1 (http://www.r-project.org/) and Bioconductor version 3.12 (http://bioconductor.org/).

## Results

### High Heterogeneity is Associated With Decreased Patient Survival in Melanoma

**Figure 1:**
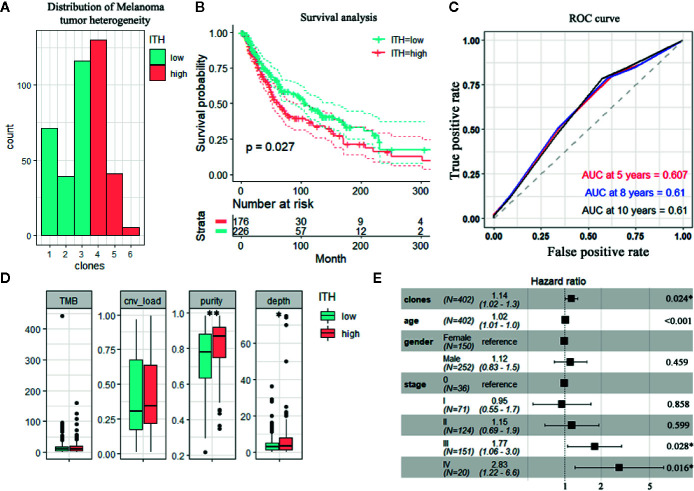
High heterogeneity is associated with decreased patient survival in TCGA melanoma cohort. **(A)** Distribution of the overall intratumor heterogeneity estimated by CHAT algorithm (n = 71, 39, 136, 130,41,9). **(B)** Kaplan–Meier analysis for overall survival rate of patients. Log-rank test was performed to evaluate the survival differences. **(C)** The prediction performance of CHAT clones for 5-, 8- and 10-year overall survival by the ROC analysis. **(D)** Boxplots correlating CHAT with purity and Breslow thickness depth. **(E)** Multivariable Cox regression analysis of the CHAT clones.

Patients were grouped according to the level of ITH that was defined by the number of clones. The clone number was computed based on each sample’s somatic copy number alterations and somatic mutations (**Figure 1A**). Patients were divided into low and high groups based on the average number of clones (CHAT high, n = 176; CHAT low, n = 226). High CHAT tumors exhibited a higher tumor purity estimation and Breslow depth, but no correlation with mutation and copy number variation (CNV) burden (**Figure 1D**). Patients with low ITH presented a significantly increased overall survival (p = 0.027; **Figure 1B**), consistent with previous observations, where patients with highly heterogeneous cancers had a reduced survival rate in pan-cancer analysis (21, 22). Receiver Operating Characteristic (ROC) analysis was performed to describe the sensitivity and specificity of survival prediction. The area under the curve (AUC) was 0.61 (**Figure 1C**). These observations remained unchanged after the integration of potential confounding factors, such as age, gender and tumor stage (**Figure 1E** and **Supplementary Figure 1**). Taking together, these results demonstrate that ITH of melanoma influences the patient`s survival rate.

### Melanoma With High Heterogeneity Are Associated With Reduced Immune Cell Infiltration

**Figure 2:**
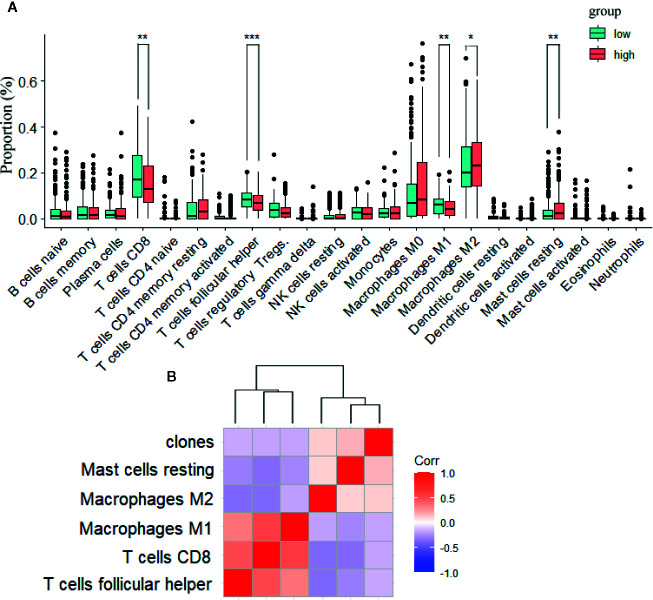
High heterogeneity is associated with reduced anti-tumor immune cell infiltration. **(A)** Cell composition fractions comparison containing 22 immune cell types between ITH high and low groups in the TCGA cohort by Wilcoxon’s test. **(B)** Correlation analysis among M1, M2, Tfh cell, resting Mast cell, CD8+T cell and clones by Spearman test. Clones is positively correlated with resting Mast cells, M2 and negatively correlated with M1, CD8+T cells, Tfh cell. *p < 0.05; **p < 0.01.

Previously, it has been shown that patients with more tumor infiltrating lymphocytes (TILs) present improved recurrence-free survival and overall survival when compared to patients with non-brisk and absent TILs (23). Therefore, we characterized the intratumoral immune landscapes within the TCGA-Melanoma cohort, and analyzed their associations with tumor heterogeneity. Each immune cell fraction calculated by CIBERSORT algorithm was compared within tumors with different CHAT clones. High CHAT tumors were associated with lower fractions of activated CD8+ T cells (p = 0.0049), follicular helper T cell (Tfh; p = 0.00091), and pro-inflammatory M1-like macrophages (p = 0.0028) as well as with higher fractions of alternatively activated M2-likemacrophages (p = 0.02; **Figures 2A, B**). In accordance with involvement of immune cells as playing in the multifactorial manifestation of tumor heterogeneity (24), we found that tumors with high heterogeneity not only possess less anti-tumor immune cells but also more immune suppressing cells.

### Tumor Heterogeneity Is Associated With Immunomodulator Gene Expression

**Figure 3:**
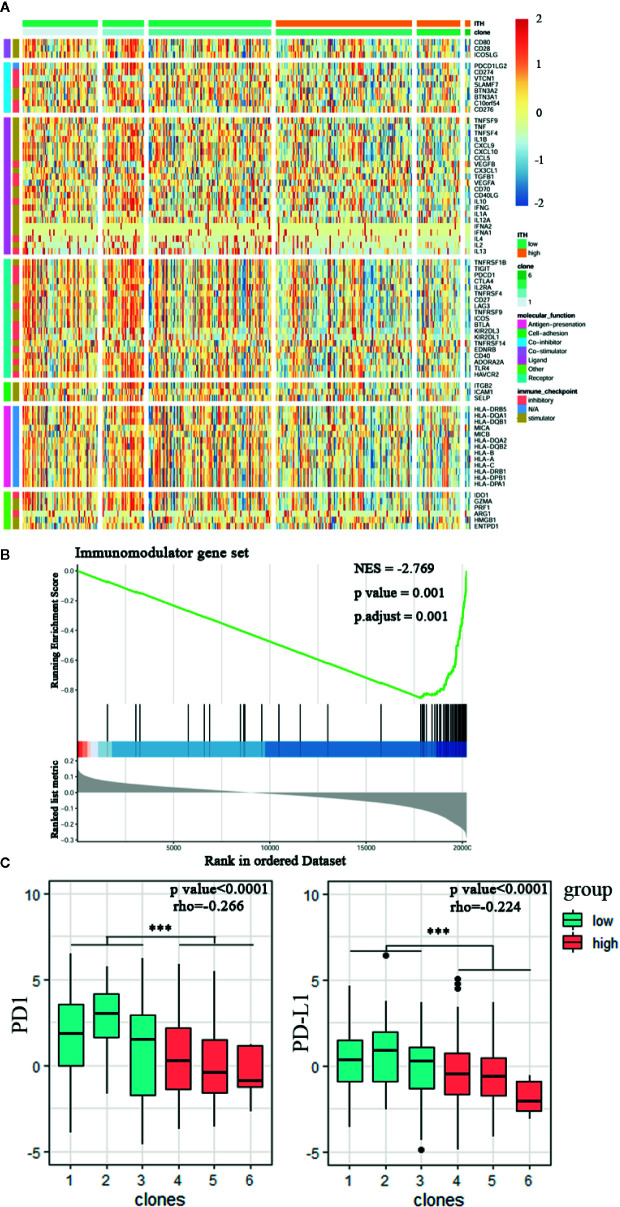
Tumor heterogeneity is associated with the regulation of Immunomodulator. **(A)** Heatmap of immunomodulator genes among the CHAT 6 clones. Data normalized by Z score transformation is used. **(B)** The gene set enrichment analysis (GSEA) of immunomodulator genes (NES = −2.769; p = 0.001). **(C)** PD1 and PD-L1 expression sectioned by clonal subclasses (Student's t test,***p < 0.001. spearman’s rho = −0.266; p <0.0001 and spearman’s rho = −0.224; p <0.0001).

Patients with distant metastatic diseases have a poor overall survival rate and respond less to conventional chemotherapy (25). Therefore, the use of immunomodulators is critical for cancer immunotherapy especially in non-responders. Nowadays, numerous immunomodulator agonists and antagonists are under investigation in clinical oncology (24). Still further progress is needed to fully evaluate their potential, understand their expression pattern, and mode of action in different stages of clonal diversity. Our examinations have identified a variation in the gene expression of immunomodulators across the 6 clonal subclasses, which might explain the role of intratumor heterogeneity in shaping the tumor microenvironment (**Figure 3A**). What’s more, the normalized enrichment score (NES) in GSEA analysis of immunomodulator gene set is −2.769(p = 0.001, **Figure 3B**). Blocking the interaction between the programmed cell death (PD)-1 protein and one of its ligands, PD-L1, has been reported to have impressive antitumor responses (26). We found that high CHAT tumor was associated with signiﬁcantly lower mRNA expression of PD1 (p = 5.6e−06, Spearman’s rho = −0.266) and PD-L1 (p = 6.2e−08, Spearman’s rho = −0.224, **Figure 3C**).

### Tumor Heterogeneity Is Associated With Immune Cytolytic Activity

**Figure 4:**
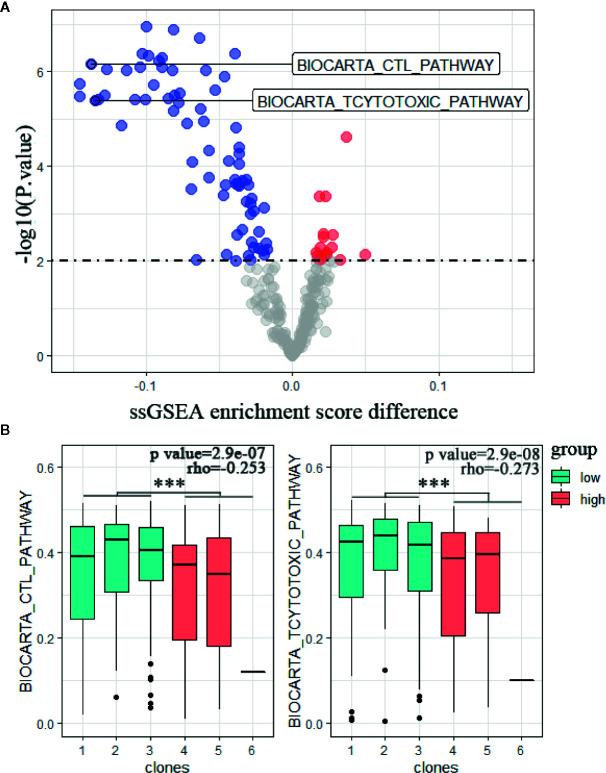
Tumor heterogeneity is associated with less immune cytolytic activity. **(A)** Volcano plot of ssGSEA enrichment score difference (x-axis) versus –log10 p value (y-axis) of BIOCARTA pathway changes in the TCGA cohort. **(B)** TCYTOTOXIC Pathway and CTL Pathway scores sectioned by clonal subclasses (Student's t test,***p < 0.001. spearman’s rho = −0.253; p <0.0001 and spearman’s rho = −0.273; p <0.0001).

The relation between the activation of the immune response and tumor heterogeneity was further investigated by scoring the enrichment of cytolytic immune pathways. Indeed, CTL score and T cytotoxic score were significantly lower in high CHAT tumors consistent with reduced cytolytic immune activity (p = 6.8E−07 and p = 1.2E−06; **Figure 4A**). Specifically, these two pathway scores were inversely correlated with the clonal diversity throughout the TCGA cohort (Spearman’s rho = −0.253, p = 2.9e−07; Spearman’s rho = −0.273, p = 2.9e−08; **Figures 4B, C**). This finding further supports the notion that reduced anti-tumor immune cell infiltration and diminished cytolytic activity within the tumor is inducing clonal evolution and the development of intratumor heterogeneity (27).

### Tumor Heterogeneity Is Associated With Immune-Related Proteins

**Figure 5:**
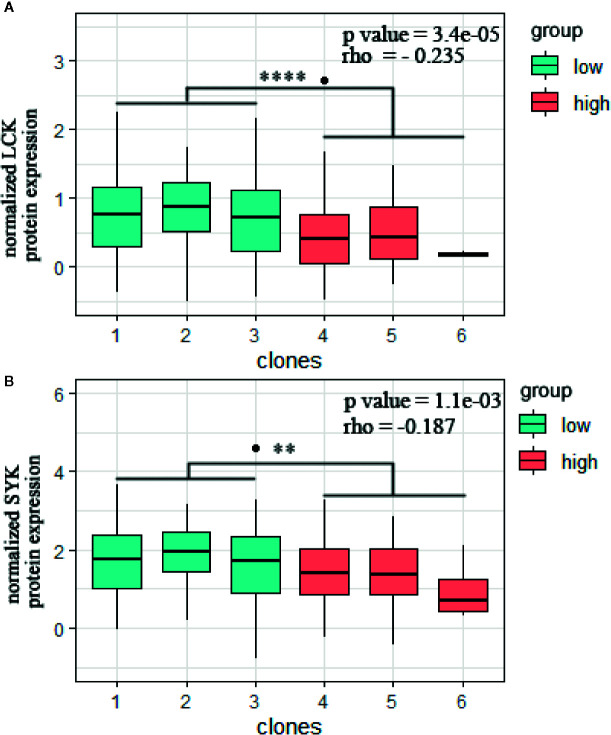
Tumor heterogeneity is associated with immune-related proteins. **(A)** LCK and **(B)** SYK normalized protein expression in RPPA array are subdivided by clonal subclasses (Student's t test, **p < 0.01; ****p < 0.0001. spearman’s rho = −0.235; p <0.0001 and spearman’s rho = −0.187; p = 0.0011).

Next, we hypothesized that the immune-related transcriptomic features, defined by the heterogeneity subclasses, are also correlated at protein levels by reverse phase protein lysate microarray (RPPA). Indeed, high ITH tumors were associated with significantly lower expression of lymphocyte-specific protein tyrosine kinase (LCK) (p = 2.3e−05, **Figure 5A**) and spleen tyrosine kinase (SYK) (p = 0.0023, **Figure 5B**) in melanoma cohort. Both proteins are non-receptor tyrosine kinases, commonly associated with anti-tumor lymphocyte signaling (28, 29). Notably, the LCK and SYK levels were inversely correlated with the degree of clone numbers throughout the TCGA cohort (Spearman’s rho = −0.235, p = 3.4e−05; Spearman’s rho = −0.187, p = 1.1e−03; **Figures 5A, B**).

## Discussion

Intratumor heterogeneity accounts for differences in tumor phenotype and clinical outcome among individual patients and has wide implications for predictive or prognostic biomarker strategies (15). In addition, tumor infiltrating lymphocytes have been identified as a marker of favorable prognosis across variety of cancers, including breast, ovarian, and colon cancer (30–32). TILs could be served as a surrogate indicator for the strength of the host anti-tumor immune response. Although multiple studies have assessed the prognostic value of tumor-infiltrating lymphocytes and immune responses in primary melanoma (33–35), the relationship between intratumor heterogeneity and immune microenvironment is still unclear. Here, we could show that patients with high tumor heterogeneity possess reduced anti-tumor immune cell infiltration, immunomodulator gene expression, and low cytolytic activity, associated with a worse survival outcome.

CHAT algorithm was used to subdivide patients into low and high score groups based on a quantitative assessment of their intratumor heterogeneity. The survival analysis in this study is in line with a previous report about breast cancer, where patients with high heterogeneity showed a tendency toward worse survival rate (36, 37). High CHAT tumors demonstrated a higher tumor purity estimation and a higher Breslow thickness depth, but no correlation with mutation burden and CNV burden, which might suggest that tumors with high heterogeneity have a higher proliferation capacity. The high heterogeneity tumors were correlated with a lower proportion of anti-tumor CD8 T cells, Tfh cells, and M1-like macrophages, whereas tumor-promoting M2-like macrophages were increased. This observation is in agreement with previous reports, showing that CD8 T cells, Tfh cells and pro-inflammatory macrophages are associated with favorable prognosis in invasive melanoma and are potential targets of immune therapies (38–41).The association between the amount of infiltrating immune cells and the degree of tumor heterogeneity could be an explanation for the observed survival differences between the compared ITH groups.

The list of immunomodulator genes was collected from a literature review performed by an interdisciplinary expert group for immune-oncology from TCGA program and the immunomodulatory function is confirmed after reviewing each entry (11). Here, we report for the first time that tumors with high heterogeneity present less gene expression of immunomodulators, which might be important for the anti-tumor immune response. The recent unprecedented success of immune checkpoint blockade that target PD-1/PD-L1 axis highlights the universal power of treating the immune system across tumor types, which offers the paradigm for scientific translation from bench to bedside (42). Nevertheless, many patients are still refractory to anti-PD-1/PD-L1 therapy. Heterogeneity is one important potential factor affecting the effectiveness of PD-1/PD-L1 blockade in melanoma. These immunomodulator genes largely contribute to the clonal variation of tumors, revealing their importance in shaping the tumor microenvironment.

Tumors with high T cell-related immune activity are associated with fewer distant metastasis, and are predictive of a higher likelihood of a complete pathologic response with chemotherapy (43). Cytotoxic T cells are key players in the cellular immune response against tumors, killing degenerated cells that display foreign antigens on their surface. The CTL pathway and the cytotoxic pathway are the main paths considered in this process, as outlined in the Molecular Signatures Database (44). Our findings suggest that tumors with high intratumor heterogeneity correlate with less immune cell infiltration, which was further supported by the demonstration of less cytolytic activity in these tumors, as shown by CTL pathway and cytotoxic T cell pathway analysis.

To confirm the correlation between tumor heterogeneity and immune activity on protein levels, the RPPA data was grouped according to the clonal subclasses. We could identify a reduced expression of the kinases LCK and SYK. LCK is one of the key molecules regulating T-cell responses in cancer immunotherapy (29). SYK, another protein tyrosine kinase, has been proven to suppress malignant growth of human breast cancer cells *via* activation of the anti-tumor immune system (28).Their high expression in tumors with low heterogeneity likely suggests a strong immune response induced by the aggregation of immune cells within the tumor tissue.

Our study provides a novel insight into the correlation of heterogeneity with prognosis, and allows for a better understanding for improving the survival of patients with melanoma. However, the limitations should be acknowledged in our study. We have no experimental data and lack clues on the link between tumor heterogeneity and the efficiency of immunotherapy such as PD-1 blockade, adoptive T cell therapy. Experimental studies are greatly needed to provide important information to strength the mechanism behind it in melanoma.

## Conclusion

Tumors with high intratumor heterogeneity demonstrate reduced infiltration with anti-tumor immune cells, lower expression of genes related to immunomodulators, lower cytolytic activity, and worse survival in melanoma. There is an urgent need to investigate the cellular, molecular and genetic changes that cause intratumor heterogeneity and it will help us to accelerate the development of prevention and treatment options, thereby improving the prognosis for melanoma patients.

## Data Availability Statement

The datasets presented in this study can be found in online repositories. The names of the repository/repositories and accession number(s) can be found below: The Cancer Genome Atlas, https://portal.gdc.cancer.gov/projects/TCGA-SKCM.

## Author Contributions

LZ, XM, and JW designed the study, performed the analyses. LZ, KK, SC, CX, and AB wrote the manuscript. JC provided expertise and input. XC, GS, and AB acquired funding, designed the study, and wrote the manuscript. All authors contributed to the article and approved the submitted version.

## Conflict of Interest

The authors declare that the research was conducted in the absence of any commercial or financial relationships that could be construed as a potential conflict of interest.
