# Supplementary material for: Intratumor Heterogeneity Correlates With Reduced Immune Activity and Worse Survival in Melanoma Patients
Source: Front Oncol. 2020 Dec 4;10:596493. doi: 10.3389/fonc.2020.596493 (PMC7747763; doi:10.3389/fonc.2020.596493)
Supplement: Supplementary file 1 [file Image_1.pdf]

Supplementary figure1

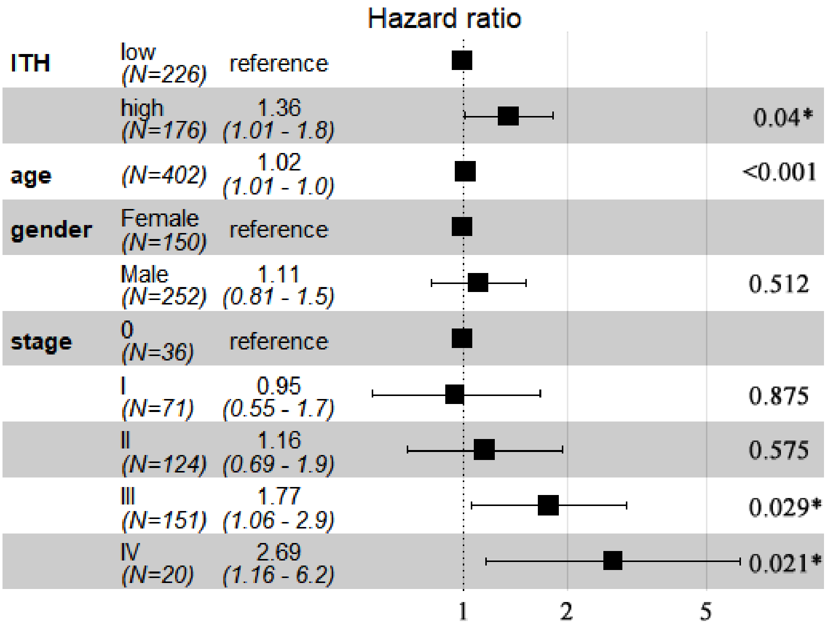

S. Figure1: Multivariable Cox regression analysis of the CHAT two groups and known prognostic clinical factors, including age at diagnosis, gender and TNM stage.
